# Case Report: Voglibose-induced persistent leukopenia in a patient with type 2 diabetes

**DOI:** 10.3389/fendo.2026.1839471

**Published:** 2026-06-04

**Authors:** Jing-wen Guo, Jin-hong Yan, Li-shuang Chang, Shuang Cai, Ya-qiu Jiang, Fen-qin Chen

**Affiliations:** 1 Department of Pharmacy, the First Hospital of China Medical University, Shenyang, Liaoning, China; 2 Department of Geriatrics, the First Hospital of China Medical University, Shenyang, Liaoning, China; 3 College of Clinical Pharmacy, Shenyang Pharmaceutical University, Shenyang, Liaoning, China

**Keywords:** adverse drug reaction, case report, leukopenia, type 2 diabetes mellitus, voglibose

## Abstract

**Aims:**

While alpha-glucosidase inhibitors like voglibose are generally considered safe, hematological adverse effects such as leukopenia are exceedingly rare. This case report investigates a potential causal link between long-term voglibose use and persistent leukopenia in a diabetic patient, emphasizing the importance of considering drug-induced cytopenia in unexplained leukopenia.

**Materials and methods:**

We describe a 71-year-old male with well-controlled type 2 diabetes who developed leukopenia over five years. After extensive evaluation, including bone marrow biopsy showing hypoplasia, and ineffective leukopoietic treatment, a multidisciplinary review identified long-term voglibose use as a possible cause. The drug was replaced with acarbose.

**Results:**

Two weeks after switching to acarbose, the patient’s white blood cell count rose from a persistent range of 2.69–3.32 × 10^9^/L to 3.89–3.98 × 10^9^/L, without other interventions. The temporal association and a Naranjo score of 7 supported voglibose as the probable cause of leukopenia.

**Conclusions:**

This case suggests that voglibose may rarely induce chronic leukopenia, especially with prolonged use. In diabetic patients with unexplained cytopenia, clinicians may consider medication review and therapeutic substitution as a diagnostic and management strategy.

## 1 Introduction

Type 2 diabetes mellitus (T2DM) is a globally prevalent chronic metabolic disorder that generally requires lifelong pharmacological management, including oral hypoglycemic agents and insulin. Alpha-glucosidase inhibitors (AGIs) are a commonly prescribed class of oral antidiabetic drugs that lower postprandial hyperglycemia by delaying carbohydrate absorption in the intestine (1). As a second-generation AGI, voglibose is widely used due to its favorable side effect profile and good tolerability, with the most frequently reported adverse reactions being gastrointestinal, such as abdominal distension and flatulence.

Drug-induced leukopenia is a clinically significant adverse effect that can increase the risk of infections and jeopardize patient safety when severe. It has been well-documented with various medications, including certain antibiotics (2), antithyroid drugs (3), and antipsychotic agents (4). However, owing to their established safety profile, AGIs—particularly voglibose—rarely include warnings about hematopoietic suppression in drug labeling, and clinical reports of such associations are exceedingly uncommon.

This article presents a case of persistent unexplained leukopenia lasting for five years in a patient with T2DM who had been on long-term voglibose therapy. Despite comprehensive evaluation—including a bone marrow biopsy indicating hypoplasia—and conventional leukopoietic treatment, little improvement was observed. A multidisciplinary team eventually suspected voglibose as the probable cause. After switching to acarbose, another AGI, the patient’s white blood cell (WBC) count increased significantly. This case aims to enhance clinicians’ awareness of this rare yet potentially adverse effect of voglibose and underscores the importance of detailed medication review in cases of unexplained cytopenia.

## 2 Case description

### 2.1 Patient information

A 71-year-old Chinese male presented with a 20-year history of elevated blood glucose and a 5-year history of leukopenia.

Physical examination on admission: height 178 cm, weight 72 kg, body mass index 22.7 kg/m², waist circumference 92 cm.

Twenty years ago, the patient was found to have elevated fasting blood glucose during a routine health examination. Following an oral glucose tolerance test, he was definitively diagnosed with T2DM. He was subsequently maintained on regular oral medication including metformin 0.5 g three times daily, voglibose 0.2–0.3 mg three times daily, and repaglinide 1–2 mg three times daily. His fasting blood glucose levels fluctuated around 6 mmol/L, postprandial glucose ranged between 8–11 mmol/L, and glycated hemoglobin levels varied from 5.9% to 6.4%.

Leukopenia was first diagnosed five years ago (January 5th, 2020), with a WBC count of 3.39×10^9^/L. No specific intervention was administered at that time. Follow-up examinations two months and six months later showed WBC counts of 3.94×10^9^/L and 3.82×10^9^/L, respectively, and again no treatment was initiated.

On April 8, 2023, a repeat test revealed a WBC count of 2.96×10^9^/L. This was the first time mild anemia was identified (hemoglobin 117 g/L), which was accompanied by generalized fatigue. Investigations for potential causes such as infection and tuberculosis were conducted, but no definitive etiology was identified. Five days later, a repeat complete blood count showed a WBC count of 4.58×10^9^/L, and no treatment was given. Over the following six months, subsequent WBC measurements were 2.95×10^9^/L and 4.26×10^9^/L, respectively.

### 2.2 Diagnostic assessment

In March 2024, the patient was readmitted. Laboratory tests showed WBC counts of 2.95×10^9^/L and 3.15×10^9^/L. Leucogen therapy (20 mg, orally, three times daily) was initiated to elevate leukocyte levels; however, the response was suboptimal. Post-treatment WBC counts remained low, fluctuating between 2.77 and 3.36×10^9^/L.

**Figure 1:**
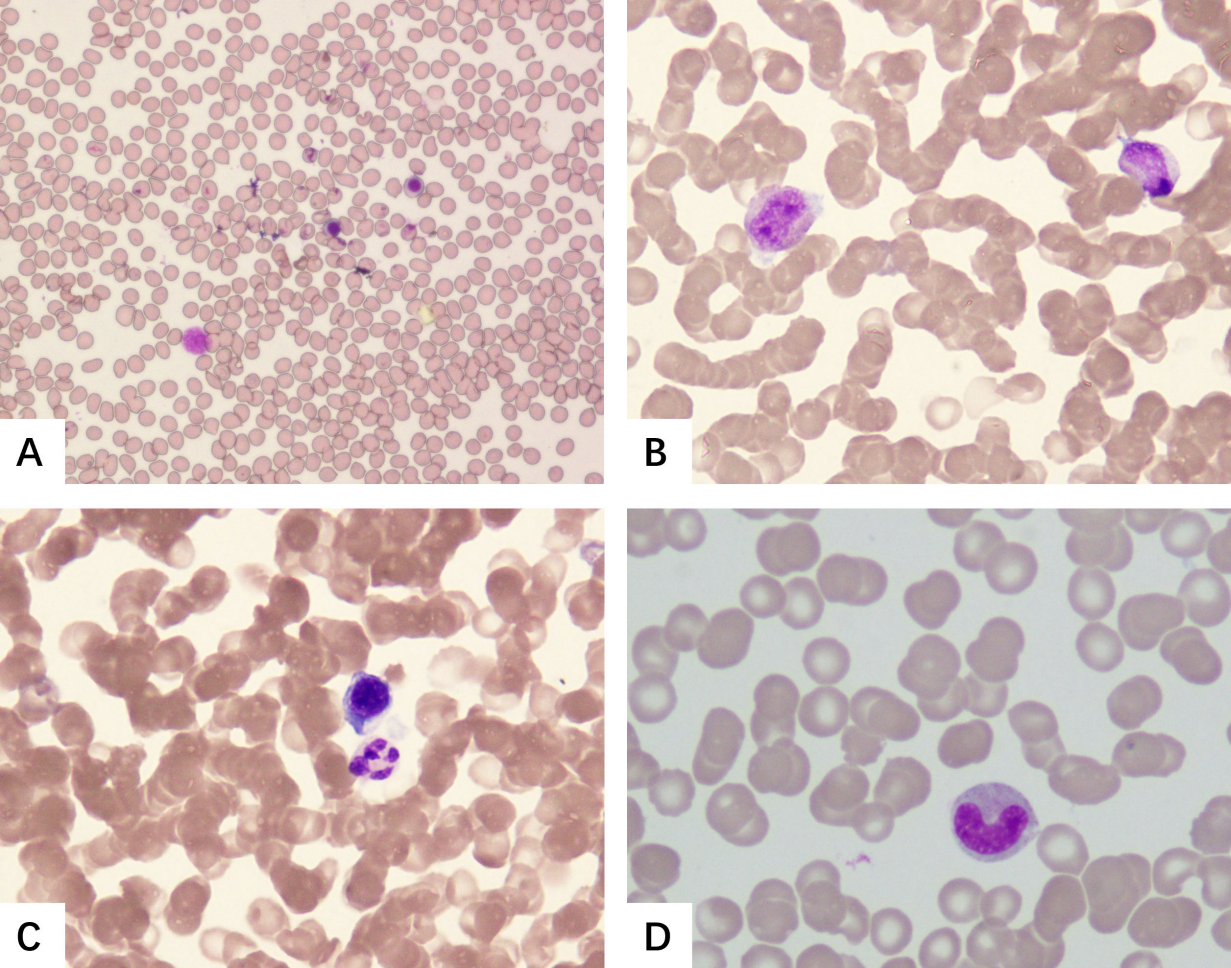
Morphology of bone marrow aspirate cells (Wright-Giemsa stain). The aspirate smear exhibits a hypocellular marrow characterized by an increased proportion of the granulocytic lineage (predominantly segmented neutrophils), a decreased proportion of the erythroid lineage, and a marked reduction in megakaryocytes. **(A)** Representative image at high magnification (x400). **(B–D)** Representative images at high magnification (x1000, oil immersion). The diagnosis of hypocellular marrow was quantitatively supported by a markedly elevated non-nucleated red blood cell to nucleated cell ratio of 200:1 (reference range: –20:1) and a significantly reduced megakaryocyte count (only 2 megakaryocytes observed per whole slide).

The patient was subsequently referred to the Department of Hematology and underwent a bone marrow aspiration on May 7, 2024 (**Figure 1**). The results revealed a hypocellular marrow with an increased granulocyte-to-erythroid ratio. Crucially, detailed morphological evaluation of the marrow smears revealed no evidence of dysplasia or dyspoiesis in any cell lineage. Furthermore, a trephine biopsy was performed, and reticulin staining was recorded as Grade MF-0, effectively ruling out bone marrow fibrosis.

He was regularly treated with leucogen (20 mg, orally, three times daily) and Shiyiwei Shenqi tablets (1.2 g, orally, three times daily), yet WBC counts remained low, ranging between 2.69–3.32×10^9^/L.

To rigorously exclude nutritional causes of leucopenia, historical records of serum vitamin B12 and folate levels were reviewed. From 2017 to 2025, serum folate levels remained consistently within the normal range (27.5–38.2 nmol/L; reference: 8.83–60.8 nmol/L), while serum vitamin B12 levels were continually supra-physiological, never falling below the normal upper limit (688.2–1281 pmol/L; reference: 145–637 pmol/L).

### 2.3 Therapeutic intervention

The patient was readmitted on July 3, 2025. A complete blood count showed a WBC level of 2.69×10^9^/L, hemoglobin of 135 g/L, and red blood cell count of 4.29×10¹²/L. Following a multidisciplinary consultation on July 8, it was recommended to discontinue voglibose and switch to acarbose. Concurrently on the same day, Shiyiwei Shenqi tablets were also discontinued to avoid confounding the observation of the hematological recovery. Metformin and repaglinide were continued at unchanged doses to serve as internal controls.

### 2.4 Follow-up and outcomes

**Figure 2:**
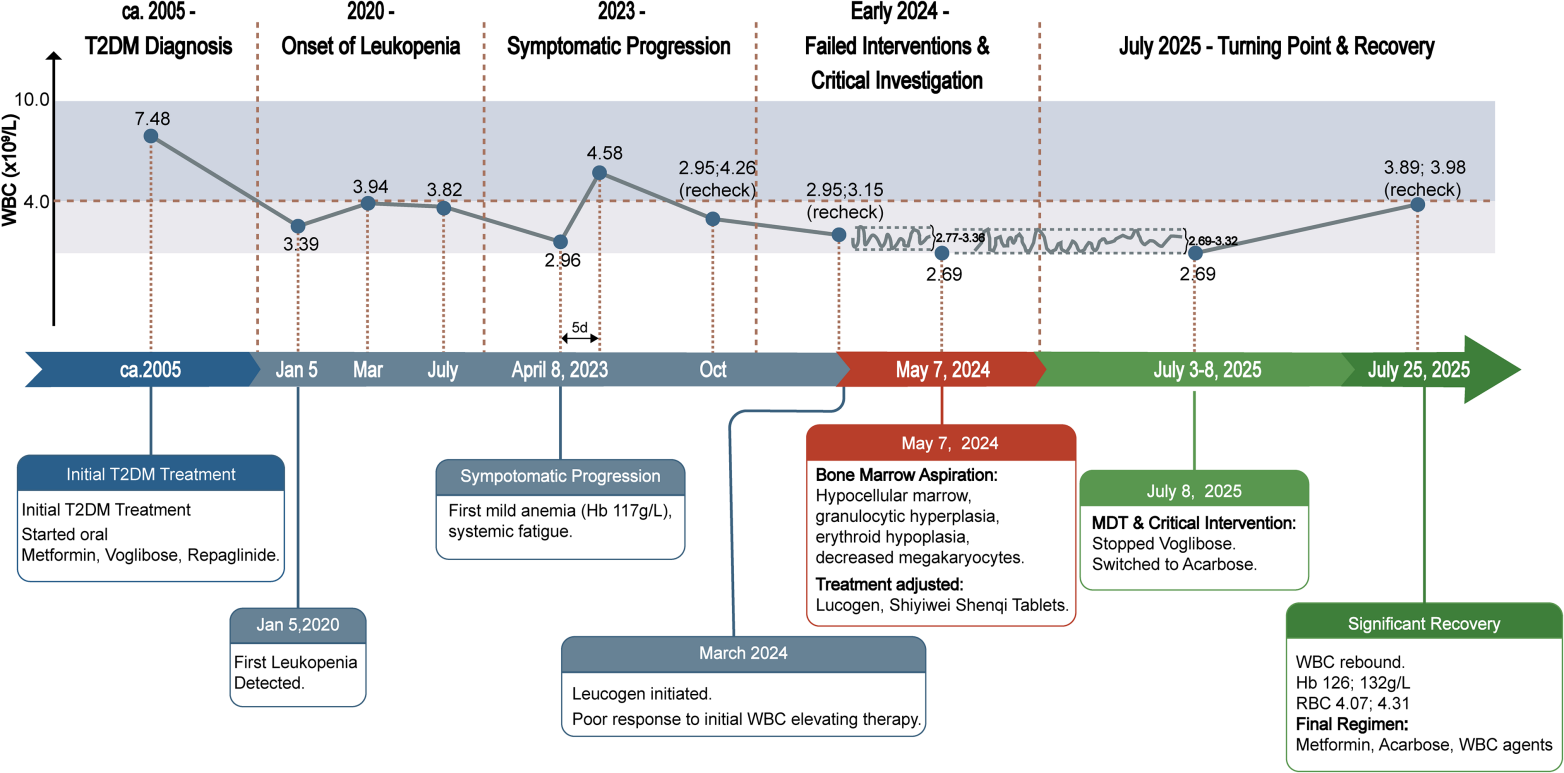
Clinical timeline and longitudinal changes in WBC count in a patient with T2DM and persistent leukopenia. WBC, white blood cell; RBC, red blood cell; Hb, hemoglobin; T2DM, type 2 diabetes mellitus; MDT, multidisciplinary team.

Two weeks later, repeat blood tests indicated an increase in WBC to 3.89×10^9^/L and 3.98×10^9^/L, hemoglobin levels of 126 g/L and 132 g/L, and red blood cell counts of 4.07×10¹²/L and 4.31×10¹²/L, respectively (**Figure 2**). The patient’s fatigue markedly improved, and glycemic control remained stable with acarbose.

His current diagnoses include T2DM, Diabetic Peripheral Neuropathy, and Leukopenia.

Upon discharge and during follow-up, his therapeutic regimen consisted of continued oral metformin and acarbose for glycemic control, along with leukopoietic agents (Leucogen and Shiyiwei Shenqi Tablets) for leukopenia.

## 3 Discussion

### 3.1 Causality assessment

Drug-induced leukopenia has been associated with various medications, including antineoplastic drugs (5), nonsteroidal anti-inflammatory drugs (NSAIDs) (6), antibacterial agents (7), antituberculosis drugs (8), antithyroid medications (3), antimalarial drugs (9), antiepileptic drugs (10), and immunosuppressive agents (11). Voglibose-induced leukopenia is a rare adverse reaction with no previously documented cases in the published literature. The product label for voglibose lists granulocytopenia (frequency unknown), anemia (0.1%–5%), and thrombocytopenia (<0.1%) as hematologic adverse reactions.

Therefore, in the differential diagnosis of unexplained leukopenia, the possibility of voglibose-induced leukopenia and its potential long-term risks should be considered, especially in patients on long-term therapy. Clinically, patients with mild leukopenia may be asymptomatic (12), whereas those with moderate to severe reductions are at significantly increased risk of infection (13) and may present with non-specific symptoms such as fatigue, dizziness, and decreased appetite (14).

Bone marrow aspiration revealed hypocellular marrow without evidence of malignancy or dysplasia. The patient’s initial WBC count prior to voglibose therapy was 7.48×10^9^/L. However, after 15 years of treatment, his WBC count first declined to 3.39×10^9^/L. With continued use exceeding 20 years, it reached a nadir of 2.69×10^9^/L. Seventeen days after voglibose was discontinued, the WBC count increased to 3.89×10^9^/L. Following a switch to acarbose, the WBC count has remained within the normal range during two subsequent outpatient follow-ups. According to a score of 7 on the Naranjo scale (15), voglibose was assessed as the probable cause of the leukopenia (**Table 1**).

**Table 1:** The Naranjo adverse drug reaction probability scale.

Question	Response	Score
1. Are there previous conclusive reports on this reaction?	Yes	1
2. Did the adverse event appear after the suspected drug was administered?	Yes	2
3. Did the adverse reaction improve when the drug was discontinued or a specific antagonist was administered?	Yes	1
4. Did the adverse event reappear when the drug was re-administered?	Do Not Know	0
5. Are there alternative causes (other than the drug) that could on their own have caused the reaction?	No	2
6. Did the reaction reappear when a placebo was given?	Do Not Know	0
7. Was the drug detected in blood (or other fluids) in concentrations known to be toxic?	Do Not Know	0
8. Was the reaction more severe when the dose was increased or less severe when the dose was decreased?	Do Not Know	0
9.Did the patient have a similar reaction to the same or similar drugs in any previous exposure?	Do Not Know	0
10.Was the adverse event confirmed by any objective evidence?	Yes	1
Total score		7

The Naranjo Adverse Drug Reaction Probability Scale evaluates the likelihood that an adverse event is related to a specific drug. The total score is interpreted as follows: ≥9 (Definite); 5 to 8 (Probable); 1 to 4 (Possible); ≤0 (Doubtful). In this case, the total score of 7 indicates a “Probable” adverse drug reaction to voglibose.

Taken together, the patient’s leukocyte levels during voglibose therapy and the Naranjo scale score indicate that the leukopenia was probably related to voglibose.

### 3.2 Alternative causes and exclusion of confounders

Given the patient’s long-term metformin use (over 20 years), vitamin B12 deficiency was an important consideration. However, the robust longitudinal laboratory data confirming continually elevated serum vitamin B12 and normal folate levels, combined with a stable mean corpuscular volume (88 fL) and the absence of neurological symptoms, completely exclude megaloblastic anemia or subclinical vitamin deficiency as the underlying etiology for the leucopenia. Furthermore, it is important to address the concurrent use and discontinuation of the herbal medicine Shiyiwei Shenqi tablets. While the simultaneous cessation of two medications on July 8, 2025, could theoretically confound the causality assessment, Shiyiwei Shenqi is a leucopoietic agent initiated years after the onset of leucopenia. Its withdrawal would logically be expected to lower, rather than elevate, the WBC count. The rapid normalization of leucocytes despite the cessation of this supportive therapy provides compelling counter-evidence against the herbal medicine being a causative confounder, thereby strongly reinforcing the primary causal role of voglibose.

To systematically evaluate other potential pharmacological etiologies, repaglinide-induced leucopenia was initially considered. However, this was definitively excluded because repaglinide was continued at an unchanged dose during the multidisciplinary intervention. The rapid normalization of the patient’s leucocyte count despite ongoing exposure to repaglinide served as a robust internal control. Furthermore, a primary hematological malignancy, specifically myelodysplastic syndrome (MDS), was systematically evaluated. The bone marrow aspirate revealed a hypocellular marrow but showed a complete absence of morphological dysplasia or dyspoiesis in any cell lineage, which strongly argues against an underlying MDS.

Finally, autoimmune phenomena and occult infections were rigorously excluded. Extensive laboratory testing revealed completely normal inflammatory markers (Erythrocyte Sedimentation Rate: 3 mm/h; C-reactive protein: < 1.0 mg/L). Although the patient had an isolated positive antinuclear antibody (ANA) finding, a comprehensive panel of specific autoantibodies (including anti-dsDNA, anti-Sm, and anti-SSA) and rheumatoid factor were entirely negative, and he lacked any systemic clinical manifestations. Most importantly, the rapid normalization of the leucocyte count strictly following voglibose withdrawal—without any concurrent immunosuppressive or antimicrobial therapy—definitively excludes both autoimmune disease and occult infection as underlying causes.

### 3.3 Mechanistic hypothesis

Drug-induced leukopenia can be categorized into two types based on its pathogenesis (16, 17): direct bone marrow suppression and immune reactions (hypersensitivity). The precise mechanism of voglibose-induced leukopenia remains unknown and the following constitutes strictly a theoretical hypothesis.

Although voglibose is a small molecule (267 Da) with minimal systemic absorption, we hypothesize that any absorbed fraction might potentially exert off-target effects. Theoretically, it could inhibit lysosomal alpha-glucosidase in hematopoietic cells—an enzyme vital for glycogen metabolism and autophagy (18–20). Impaired autophagy might subsequently disrupt hematopoietic stem cell homeostasis, potentially contributing to a hypocellular marrow and peripheral cytopenia. However, it is crucial to emphasize that this pathway is entirely speculative. There is currently no direct evidence linking voglibose exposure to its intracellular accumulation in bone marrow cells. Furthermore, no specific laboratory investigations—such as intracellular drug level monitoring, *in vitro* cellular assays, or genetic testing—were performed in this study to corroborate this proposed mechanism. In contrast, acarbose (645 Da) is a larger molecule and theoretically less likely to enter cells or inhibit intracellular alpha-glucosidase, which might explain its safety as a therapeutic substitute in this patient.

### 3.4 Strengths and limitations

This case has several strengths, including a long observation period, detailed bone marrow findings, and a well-documented drug challenge-dechallenge sequence with internal controls. However, limitations must be acknowledged. First, the herbal medicine Shenqi Shiyiwei tablets were used concurrently during part of the treatment course. While the leukopenia persisted despite its use, we cannot entirely rule out its potential influence on the recovery phase. Second, re-challenge with voglibose was not performed due to safety and ethical concerns, which limits the ability to confirm causality definitively. Third, as a single case report, the findings may not be generalizable to other patient populations. Finally, the proposed mechanism of lysosomal AGI remains speculative and requires further laboratory investigation.

### 3.5 Take-away lessons

This case suggests the critical need to recognize drug-induced leukopenia, particularly in patients presenting with unexplained leukopenia or those undergoing long-term voglibose therapy for diabetes. Early identification and intervention can markedly enhance clinical outcomes. In clinical practice, healthcare providers often prioritize inadequate disease control or primary hematologic disorders, potentially overlooking drug-induced leukopenia as an etiology. Multidisciplinary teams offer distinct advantages by fostering collaborative diagnosis and treatment among specialists, who actively contribute to rational pharmacotherapy and adverse drug reaction monitoring, evaluation, and management. Such an integrated approach is instrumental in minimizing patient harm and ensuring medication safety. However, it must be noted that the substitution of voglibose with acarbose in this context has not been systematically studied in larger populations, and such a change requires close hematological and glycaemic monitoring.

## 4 Patient perspective

The following perspective was obtained via a semi-structured interview with the patient in his native language. The authors translated and summarized the patient’s narrative, and the final English text was explained to and approved by the patient to ensure it accurately reflects his experience.

I have had T2DM for 20 years and adhered to regular oral hypoglycemic therapy with well-controlled blood glucose. I was surprised to be diagnosed with leukopenia in a routine check-up five years ago, and grew anxious as my WBC count fluctuated and declined, accompanied by persistent fatigue that disrupted daily life. I completed all recommended examinations including bone marrow aspiration and took prescribed leukopoietic drugs, but saw little improvement in my blood indices, which left me worried about the unknown cause and potential risks.

I fully trusted the multidisciplinary team’s advice to switch voglibose to acarbose. I was greatly relieved that my WBC count rose to the normal range just two weeks after the medication change, with no new discomfort and stable blood glucose. I am grateful for the team’s careful diagnosis and timely intervention. This experience has made me recognize the importance of regular check-ups and close doctor communication during long-term chronic disease medication, and I will strictly follow medical advice for future follow-ups and treatment management.

## Data Availability

The original contributions presented in the study are included in the article/supplementary material. Further inquiries can be directed to the corresponding authors.

## References

[1] Moritoh Y Takeuchi K Hazama M . Chronic administration of voglibose, an alpha-glucosidase inhibitor, increases active glucagon-like peptide-1 levels by increasing its secretion and decreasing dipeptidyl peptidase-4 activity in ob/ob mice. J Pharmacol Exp Ther. (2009) 329:669–76. doi: 10.1124/jpet.108.148056. PMID: 19208898

[2] de Jonge NA Sikkens JJ Zweegman S Beeker A Ypma P Herbers AH . Short versus extended treatment with a carbapenem in patients with high-risk fever of unknown origin during neutropenia: a non-inferiority, open-label, multicentre, randomised trial. Lancet Haematol. (2022) 9:e563–72. doi: 10.1016/s2352-3026(22)00145-4. PMID: 35691326

[3] Kamitani F Nishioka Y Koizumi M Nakajima H Kurematsu Y Okada S . Antithyroid drug-induced leukopenia and G-CSF administration: a long-term cohort study. Sci Rep. (2023) 13:19336. doi: 10.1038/s41598-023-46307-5. PMID: 37935745 PMC10630492

[4] Oloyede E Dunnett D Taylor D Clark I MacCabe JH Whiskey E . The lived experience of clozapine discontinuation in patients and carers following suspected clozapine-induced neutropenia. BMC Psychiatry. (2023) 23:413. doi: 10.1186/s12888-023-04902-w. PMID: 37291505 PMC10249299

[5] Yu Z Daihong G Chong Y Hongyi Y Chengxuan Y Siyuan L . Analysis of 2808 cases of spontaneous report associated with drug - inducedleukopenia. Vet Clin Pathol. (2020) 36:1719–22.

[6] Chen-Goodspeed A Jonas N Merola S Sample J . Perforated duodenal ulcer 24 years after Roux-en-Y gastric bypass: a rare presentation with pneumoperitoneum. Cureus. (2025) 17:e82107. doi: 10.7759/cureus.82107. PMID: 40351907 PMC12066166

[7] Han H Yan H King KY . Broad-spectrum antibiotics deplete bone marrow regulatory T cells. Cells. (2021) 10:277. doi: 10.3390/cells10020277. PMID: 33573218 PMC7911786

[8] Lin FS Wu MY Tu WJ Pan HQ Zheng J Shi JW . A cross-sectional and follow-up study of leukopenia in tuberculosis patients: prevalence, risk factors and impact of anti-tuberculosis treatment. J Thorac Dis. (2015) 7:2234–42. doi: 10.3978/j.issn.2072-1439.2015.12.41 PMC4703664 26793345

[9] Stürchler D Schär M Gyr N . Leucopenia and abnormal liver function in travellers on malaria chemoprophylaxis. J Trop Med Hyg. (1987) 90:239–43. doi: 10.1007/978-1-60327-297-1_20. PMID: 3669125

[10] Dohány A Guija-de-Arespacochaga A Fux D Silberbauer C Pákozdy Á . A retrospective evaluation of phenobarbital-induced hematologic changes in 69 cats. Vet Clin Pathol. (2023) 52:601–6. doi: 10.1111/vcp.13259 37721182

[11] Jung HY Lee S Jeon Y Choi JY Cho JH Park SH . Mycophenolic acid trough concentration and dose are associated with hematologic abnormalities but not rejection in kidney transplant recipients. J Korean Med Sci. (2020) 35:e185. doi: 10.3346/jkms.2020.35.e185. PMID: 32567256 PMC7308135

[12] Lihua Z . Clinical pharmacological analysis of vancomycin and norvancomycin-induced leukopenia. Chin J Clin Rational Drug Use. (2017) 10:76–7.

[13] Badawy SM Palmblad J Tricta F Toiber Temin N Fradette C Lin L . Rates of severe neutropenia and infection risk in patients treated with deferiprone: 28 years of data. Blood Adv. (2024) 8:5641–9. doi: 10.1182/bloodadvances.2023012316. PMID: 38640437 PMC11565021

[14] Crawford J Herndon D Gmitter K Weiss J . The impact of myelosuppression on quality of life of patients treated with chemotherapy. Future Oncol. (2024) 20:1515–30. doi: 10.2217/fon-2023-0513. PMID: 38587388 PMC11441072

[15] Naranjo CA Busto U Sellers EM Sandor P Ruiz I Roberts EA . A method for estimating the probability of adverse drug reactions. Clin Pharmacol Ther. (1981) 30:239–45. doi: 10.1038/clpt.1981.154. PMID: 7249508

[16] Fei Z Qi L Lijun H Ying H . The releasing raw of the antibiotic *in vitro* in a new norvancomycin-fibringlue calcium phosphate bone substitute. J Gannan Med Univ. (2014) 34:25–7.

[17] Yanwen Z Jie Z . Clinical pharmaceutical analysis of leukopenia induced by vancomycin and norvancomycin. Zhejiang Clin Med J. (2016) 18:487–8.

[18] Kuriyama C Kamiyama O Ikeda K Sanae F Kato A Adachi I . *In vitro* inhibition of glycogen-degrading enzymes and glycosidases by six-membered sugar mimics and their evaluation in cell cultures. Bioorg Med Chem. (2008) 16:7330–6. doi: 10.1016/j.bmc.2008.06.026. PMID: 18595718

[19] Tan K Tesar C Wilton R Jedrzejczak RP Joachimiak A . Interaction of antidiabetic α-glucosidase inhibitors and gut bacteria α-glucosidase. Protein Sci. (2018) 27:1498–508. doi: 10.1002/pro.3444. PMID: 29761590 PMC6153411

[20] Durga Priyadharshini R Ponkarpagam S Vennila KN Elango KP . Spectroscopic and theoretical evidences for the surface binding of voglibose drug with DNA. Spectrochim Acta A Mol Biomol Spectrosc. (2022) 271:120888. doi: 10.1016/j.saa.2022.120888. PMID: 35063822

